# Developmental milestones in type I spinal muscular atrophy

**DOI:** 10.1016/j.nmd.2016.10.002

**Published:** 2016-11

**Authors:** Roberto De Sanctis, Giorgia Coratti, Amy Pasternak, Jacqueline Montes, Marika Pane, Elena S. Mazzone, Sally Dunaway Young, Rachel Salazar, Janet Quigley, Maria C. Pera, Laura Antonaci, Leonardo Lapenta, Allan M. Glanzman, Danilo Tiziano, Francesco Muntoni, Basil T. Darras, Darryl C. De Vivo, Richard Finkel, Eugenio Mercuri

**Affiliations:** a Paediatric Neurology Unit, Catholic University and Centro Clinico Nemo, Rome, Italy; b Department of Neurology, Boston Children's Hospital, Harvard Medical School, Boston, USA; c Department of Neurology, Columbia University Medical Center, New York, USA; d Department of Physical Therapy, The Children's Hospital of Philadelphia, Philadelphia, USA; e Institute of Genetics, Catholic University, Rome, Italy; f Dubowitz Neuromuscular Centre, UCL Institute of Child Health & Great Ormond Street Hospital, London, UK; g Nemours Children's Hospital, University of Central Florida College of Medicine, Orlando, USA

**Keywords:** Spinal muscular atrophy, Hammersmith Infant Neurological Examination, Outcome measures, Motor milestones

## Abstract

The aim of this retrospective multicentric study was to assess developmental milestones longitudinally in type I SMA infants using the Hammersmith Infant Neurological Examination. Thirty-three type I SMA infants, who classically do not achieve the ability to sit unsupported, were included in the study. Our results confirmed that all patients had a score of 0 out of a scale of 4 on items assessing sitting, rolling, crawling, standing or walking. A score of more than 0 was only achieved in three items: head control (n = 13), kicking (n = 15) and hand grasp (n = 18). In these items, the maximal score achieved was 1 out of a scale of 4, indicating only partial achievement of the milestone. Infants with symptom onset after 6 months of age had longer preservation of a score of 1 when compared to those with onset before 6 months of age. Our results suggest that even when current standards of care are applied, developmental milestones are rarely even partially achieved as part of natural history in type I SMA infants. No infants in this study achieved a major milestone such as rolling over, or sitting independently, which would therefore represent robust outcomes in future interventional trials.

## Highlights

• This paper reports patterns of natural progression in type I SMA.
• The HINE is used to capture motor developmental milestones in SMA.
• Motor developmental milestones are rarely acquired in type I SMA infants.

## 1 Introduction

Spinal muscular atrophy (SMA) is an autosomal recessive motor neuron disorder caused by mutations in the survival of motor neuron 1 (SMN1) telomeric gene, resulting in a reduced amount of functional full-length SMN protein. In children, SMA is classified into 3 phenotypes based on age at onset and maximal motor function achieved [1], [2].

Type I SMA is characterized by inability to sit unsupported and by onset within 6 months of age [2], [3]. Survival, typically limited to the first year, has increased in the last few decades, due to improvements in the standards of care, with the possibility to opt for respiratory and nutritional support [4], [5].

The increased survival and the advent of different therapeutic approaches have highlighted the need for a better definition of the natural history of type I SMA especially as far as the acquisition of functional milestones is concerned, and for identification of appropriate tools to follow the progression of the disease. A few attempts have been made recently to develop clinical tools specifically designed to assess motor function in weak infants, such as the CHOP INTEND [6], or to adapt existing scales, such as the TIMP [7], [8]. These scales have helped us to identify the variability of the phenotype and to assess the rapid progression of the disease. Little attempt has been made to monitor developmental milestones in these infants as, by definition, they are unable to sit and only a minority of them will acquire the milestones of head control and/or rolling over which are subsequently lost. Other important milestones, such as crawling or standing, are not achieved in type I SMA infants. However, no systematic study has assessed longitudinally the acquisition of motor milestones in type I SMA infants receiving current standards of care.

The aim of this study was to report the longitudinal use of a module capturing developmental milestones in infancy, performed as part of the Hammersmith Infant Neurological Examination (HINE) [9], [10], in type I SMA infants. More specifically, we set out to investigate whether developmental milestones are achieved at any stage during the first years of life in these infants and, if achieved, to assess possible changes over time.

## 2 Materials and methods

This retrospective study was performed by collecting data from different existing large multicentric datasets, including one network based in the United States (the Pediatric Neuromuscular Clinical Research Network for SMA) and one in Italy, in the period between 2010 and October 2014.

All patients had a genetically confirmed diagnosis of SMA with a homozygous deletion of exon 7 in the *SMN1* gene, and a clinically confirmed diagnosis of type I SMA.

The diagnosis of SMA was made by the principal investigator at each site who also classified them according to the Dubowitz's decimal classification [11] and to the recently proposed classification in 3 subtypes [4], [12]: type IA presentation at birth with joint contractures and need for respiratory support, or onset of motor and respiratory involvement in the first week; type IB, symptom onset after neonatal period, usually before age 3 months, head control never achieved; and type IC, onset after neonatal period, usually between 3 and 6 months, head control achieved.

Previously identified patients, followed up in our clinics, and newly diagnosed patients were enrolled. All eligible patients were offered participation. We included all infants who had at least two or more assessments over the following two years.

The study was approved by the individual institutional review boards at the participating sites.

Study visits were scheduled at baseline, when first assessed, and, when possible, every 2–3 months until the age of 12 months and every 6 months thereafter, depending on the infant's health and ability to travel.

**Fig. 1:**
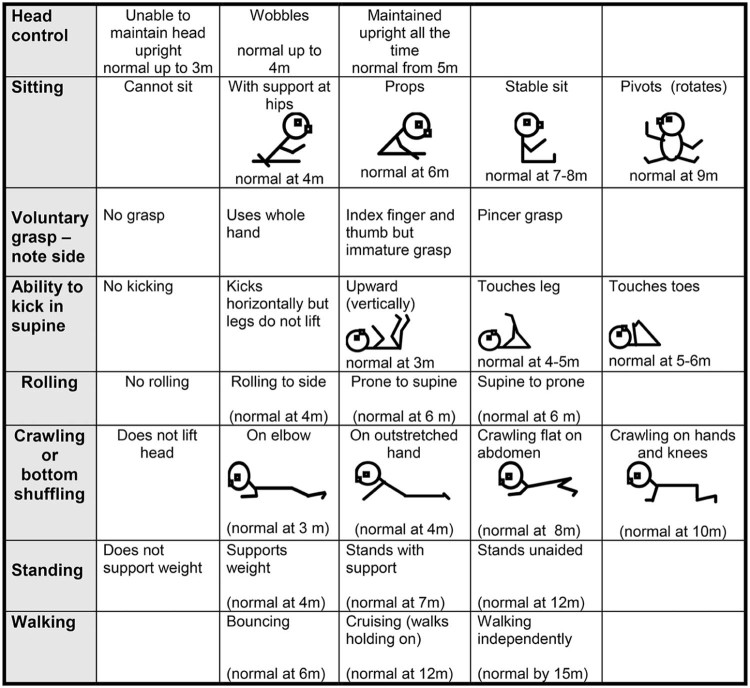
HINE scoring module illustrating the motor developmental milestones.

The HINE [9] is a simple and scorable method for assessing infants between 2 and 24 months of age, including different aspects of neurological examinations as cranial nerves, posture, movements, tone and reflexes. A separate section includes eight selected motor items which document developmental progress (Fig. 1). It provides a summary of motor developmental milestones giving not only the opportunity to record the age at which the various milestones were achieved but also allowing one to quantify intermediate steps leading to the full achievement of the milestone. Each item provides the opportunity to score the level of development on a 5 point scale with 0 as absence of the activity. These milestones were designed according to the gradient of normal maturation, with normative values adapted from Illingworth's work [13]. These items are more granular than the World Health Organization's motor milestones, which capture sitting without support, hands-and-knees crawling, standing with assistance, standing alone, walking with assistance and walking alone [14].

The HINE module, on the other hand, is shorter and includes less items of the motor component of the Bayley scales of infants and toddler development that include many items that are not relevant to type I SMA and is more fatiguing for such fragile infants.

### 2.1 Statistical analysis

The distribution of scores was plotted for individual infants for each item plotting longitudinal results according to age.

## 3 Results

Thirty-three infants fulfilled the inclusion criteria and were included in the study. Their age at onset ranged between 1 and 8 months. The age when they were first assessed was between 2 and 9 months.

Seven infants, who were at the severe end of the spectrum of weakness and had early presentation with motor and respiratory involvement within the first week, were classified as 1.1, using the Dubowitz decimal system, or as type IA according to the recent classification. Two other infants, who achieved head control, were at the other end of the spectrum, and were classified as 1.9, or type IC according to the recently proposed classification. In both, onset of signs was around 7–8 months.

Twenty-four infants had the more typical course of type I SMA, and were classified as 1.5, or type IB. In all 24 the onset was after the first week but before the age of 5–6 months. Two of these 24 achieved transient head control.

### 3.1 Motor developmental milestones

#### 3.1.1 Head control

All 7 infants at the weakest end of the spectrum had scores of 0 on all the assessments.

**Fig. 2:**
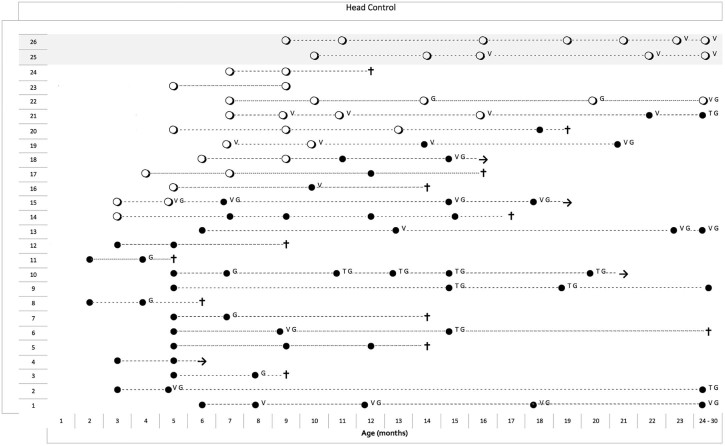
Longitudinal data of the item assessing the ability to control the head in 24 typical type I SMA infants (from number 1 to 24) and in 2 stronger type I infants with late onset (25–26, shaded). Each line represents the different assessments in the same patient linked by a dotted line. ● = score 0, ○ = score 1, V = ventilation; G = gastrostomy; T = tracheostomy). The seven weakest patients with onset within the first week (type IA or 1.1 according to Dubowitz) were not added to the figure as they all had a score of 0 in all assessments.

Among the 24 infants in the main group, 11 had a score of 1 (wobbles) on at least one assessment, 13 had a score of 0 on all assessments. None had a score of 2 or above (Fig. 2).

Infants who had a score of 0 never achieved higher scores on subsequent assessments.

The 2 stronger infants with late onset had a score of 1 that persisted throughout the assessments over the following 2 years.

#### 3.1.2 Voluntary grasp

All 7 infants at the weakest end of the spectrum had a score of 0 on all the assessments.

**Fig. 3:**
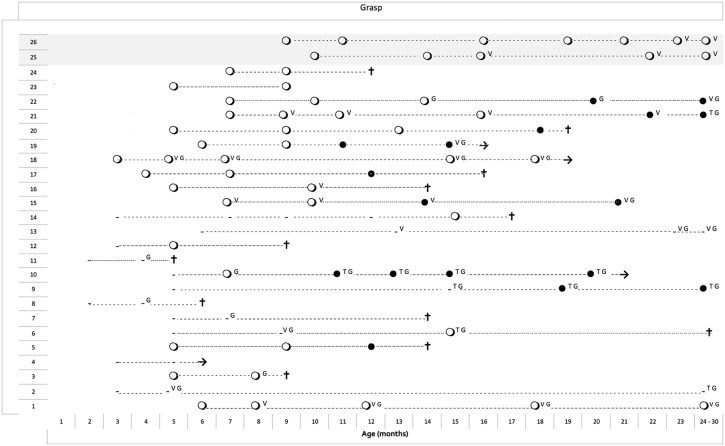
Longitudinal data of the item assessing voluntary grasp in 24 typical type I SMA infants (from number 1 to 24) and in 2 type I infants with late onset (25–26, shaded). Each line represents the different assessments in the same patient linked by a dotted line. ● = score 0, ○ = score 1, V = ventilation; G = gastrostomy; T = tracheostomy). The seven patients with onset within the first week (type IA or 1.1 according to Dubowitz) were not added to the figure as they all had a score of 0 in all assessments.

Among the 24 infants in the main group, 17 had a score of 1 (using whole hand) on at least one assessment, and 7 had a score of 0 on all assessments. None had a score of 2 or above (Fig. 3).

None of the infants who had a score of 0 on one assessment had better scores on the subsequent assessments.

The 2 stronger infants with late onset achieved a score of 1 that persisted throughout the assessments over the following 2 years.

#### 3.1.3 Ability to kick

All 7 infants at the weakest end of the spectrum had a score of 0 on all the assessments.

**Fig. 4:**
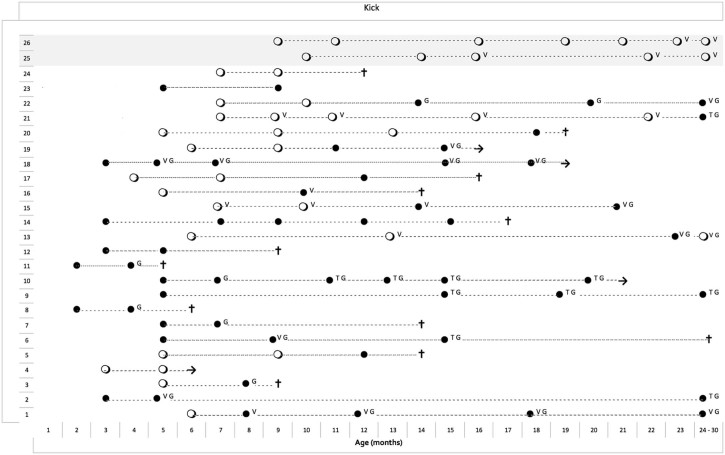
Longitudinal data of the item assessing ability to kick in 24 typical type I SMA infants (from number 1 to 24) and in 2 type I infants with late onset (25–26, shaded). Each line represents the different assessments in the same patient linked by a dotted line. ● = score 0, ○ = score 1, V = ventilation; G = gastrostomy; T = tracheostomy). The seven patients with onset within the first week (type IA or 1.1 according to Dubowitz) were not added to the figure as they all had a score of 0 in all assessments.

Among the 24 infants in the main group, 13 had a score of 1 (gravity-eliminated movements) on at least one assessment, and 11 had a score of 0 on all assessments. None had scores of 2 or above (Fig. 4). Only one of the infants who had a score of 0 achieved a score of 1 on subsequent assessments. The 2 stronger infants with late onset had a score of 1 that persisted throughout the assessments over the following 2 years.

#### 3.1.4 Other items

All infants in this study had a score of 0 on all visits in all the remaining 5 items (sitting, rolling, crawling, standing, walking).

#### 3.1.5 Gastrostomy and ventilator support

In the main group, 15 of the 24 infants underwent gastrostomy for swallowing problems and/or failure to thrive, 10 started non-invasive ventilation and 5 had tracheostomy. None had any improvement following the introduction of any of these procedures. In the few patients in whom the scores on individual items were 1 at the time these procedures were introduced, a score of 1 was not always maintained (Fig. 2, Fig. 3, Fig. 4).

## 4 Discussion

Improvements in standards of care with the more systematic use of optimal respiratory care including non-invasive ventilation and cough augmentation techniques and better management of nutritional issues have resulted in improved survival in type I SMA in the last decade. This has raised the question of whether the improved survival is also associated with possible acquisition of motor developmental milestones over time.

In order to capture possible changes in the achievement of motor developmental milestones in infancy, we used a module extracted from the HINE, an assessment of global neurological function and motor developmental milestones in infants that can be used from the age of 2–3 months. The HINE has already been used in several clinical groups [15] and has been validated in typically developing children [9]. Using the developmental module from the HINE we were able to confirm that motor developmental milestones are only rarely acquired in type I SMA infants after the diagnosis is made.

Not surprisingly, infants with the most severe phenotype who also had very early onset of clinical signs never acquired any motor developmental milestones, showing a score of 0 on all of the 8 items at all times.

In contrast, scores higher than 0 could be observed in three of the items in the cohort of infants with onset after the first week of life.

By definition, type I SMA infants are unable to sit unsupported. Our results confirmed that none of the infants achieved independent sitting nor any other of the subsequent milestones, such as crawling or standing in which all patients had a score of 0 at all times. This was also true with the 2 stronger late onset infants who were at the stronger end of the spectrum.

A score higher than 0 was only achieved in head control (n = 13), kicking (n = 15) and hand grasp (n = 18). It is of interest that even in those items, the maximal score achieved was 1 out of a scale of 4, indicating only partial achievement of the milestone.

Partial head control was only achieved in 11 infants and was reported as wobbling. This was transient in the infants in the main cohort and sustained over several assessments in the 2 stronger infants with late onset. None achieved a constant full head control for more than 15 seconds.

Thirteen infants were able to kick horizontally on at least one assessment but legs did not lift with full antigravity movements at any time, including the 2 stronger infants.

Similarly, although the item exploring hand grasp was not specifically designed to capture changes in weak infants but is mainly assessing development of fine motor skills, only a few infants achieved the ability to use the whole hand and only the 2 stronger infants developed better hand grip.

Overall, when present, a score of 1 was found in younger infants on their first assessments and, with only one exception, none showed any improvement from 0 to 1 on subsequent evaluations. Therefore, increased survival does not appear to increase the possibility of acquiring developmental milestones over time, or to improving their scores even if a full milestone is not achieved. This finding, in fact, is not surprising as several studies have reported progressive loss of motor neurons in both animal models and humans; using neurophysiological biomarkers, like CMAP and MUNE, severe and substantial postnatal progression of motor denervation has been shown in the first months postnatally in type I SMA [16].

When we looked at the possible relationship between ventilatory or nutritional support and developmental milestones, the numbers were too small to allow a meaningful statistical analysis but it was obvious that the introduction of gastrostomy or ventilator support was not consistently associated either with higher scores or with a longer persistence of a score of 1.

This may be explained by the fact that while better standards of care may facilitate well-being and increased survival, the need for ventilation or gastrostomy is, in most cases, a sign that the disease has already progressed and is less likely to be associated with subsequent improvements.

One of the limitations of the study is its retrospective nature; however, data were collected over a limited period of time in centers that were complying with current standards of care. In order to avoid possible bias, the recruitment period was also limited to the time when enrollment for clinical trials had not started in the participating centers as, after that, we would have included only the infants who, for some reason, were thought not to be eligible for clinical trials. The relative small number of patients was justified by the exclusion of infants who had only one assessment and then decided to follow palliative care locally. It is of interest, however, that infants who had only a single assessment were generally performing very poorly and none had a score of 2 (data not shown).

Our results suggest that even with current standards of care, motor developmental milestones are rarely acquired, even partially, as part of natural history in type I SMA infants. Even the stronger ones, who had longer preservation of the scores of 1 when compared to the weaker infants with a more typical course, failed to make any further gains after the first evaluation. In future drug intervention studies, type I SMA infants who achieve motor milestones beyond what is described here can be attributed, with a strong probability, as being due to drug effect and not to enhanced standard of care or prolonged survival. Further longitudinal prospective studies, exploring, in more detail, the correlation with standards of care, SMN2 copy numbers, neurophysiology or biomarkers will help to further elucidate the progression and the variability of the disease.

## Funding

This study was funded by Famiglie SMA and Telethon (Italy) (GSP 13002), the SMA Foundation (to PNCR) and SMA Trust (UK).
